# A Potential Link Between Oral Microbiota and Female Reproductive Health

**DOI:** 10.3390/microorganisms13030619

**Published:** 2025-03-07

**Authors:** Justyna Marcickiewicz, Małgorzata Jamka, Jarosław Walkowiak

**Affiliations:** Department of Pediatric Gastroenterology and Metabolic Diseases, Poznan University of Medical Sciences, Szpitalna Str. 27/33, 60-572 Poznan, Poland; just.marcickiewicz@gmail.com (J.M.); jarwalk@ump.edu.pl (J.W.)

**Keywords:** female reproductive tract, polycystic ovary syndrome, endometriosis, periodontal health, oral dysbiosis, oral microbiome, infertility

## Abstract

Oral cavity dysbiosis is associated with numerous inflammatory diseases, including diabetes, inflammatory bowel diseases, and periodontal disease. Changes in the oral microenvironment lead to bidirectional interactions between pathogens and individual host systems, which may induce systemic inflammation. There is increasing evidence linking the condition of the oral cavity with the most common causes of female infertility, such as polycystic ovary syndrome and endometriosis, as well as gestational complications, e.g., low birth weight, preterm delivery, and miscarriages. This review highlights the composition of the female oral microbiome in relation to infertility-related disorders, such as endometriosis and polycystic ovary syndrome, and provides a comprehensive overview of the current state of knowledge on the relationship between a dysbiotic oral microbiome, pregnancy, and its impact on the female reproductive tract.

## 1. Introduction

The human oral microbiome comprises colonies of microorganisms inhabiting the teeth, tongue, soft and hard palate, cheeks, tonsils, and gingival sulci [1], which begin to develop at birth and change throughout adolescence and adulthood [2]. This heterogeneous ecosystem, located in the initial section of the digestive tract, consists of approximately 1000 species of bacteria, viruses, fungi, protozoa, and archaea, with bacteria being the most numerous group [3]. The most common species in healthy humans are *Streptococcus*, *Prevotella*, *Rothia*, *Neisseria*, *Haemophilus*, *Fusobacterium*, *Corynebacterium*, *Actinomyces*, *Capnocytophaga*, *Porphyromonas*, and *Granulicatella* [4]. Moreover, individual microbial species inhabiting the oral cavity are characterized by a certain local specificity. Pioneering microorganisms first to colonize the oral cavity are *Streptococcus* species, among which *Streptococcus mitis* occurs mainly in the buccal mucosa, *Streptococcus infantis* colonizes the hard palate, *Streptococcus sanguinis* and *Streptococcus gordonii* colonize the teeth, and *Streptococcus australis* colonizes the back and lateral surface of the tongue [5,6,7,8,9]. *Gemella hemolysans* is the dominant species on the buccal epithelium, *Granulicatella elegans* is dominant on the hard palate, and the tooth surface is inhabited by *Rothia dentocariosa* [4]. All of these microorganisms together create a diverse and highly developed ecosystem.

The overgrowth of pathogenic species and the loss of microbial biodiversity disrupt the natural homeostasis, finally leading to a condition known as dysbiosis. Oral cavity dysbiosis may induce the development of numerous oral diseases, including dental caries and periodontal diseases [10]. Periodontal diseases are one of the most common inflammatory diseases among adults and are caused by, among other things, poor oral hygiene, diabetes, excessive sugar consumption, smoking, and obesity [11]. Several studies have shown that anaerobic Gram-negative bacteria, mainly *Campylobacter rectus*, *Treponema denticola* (*T. denticola*), *Actinobacillus actinomycetemcomitans* (*A. actinomycetemcomitans*), *Prevotella intermedia* (*P. intermedia*), *Porphyromonas gingivalis* (*P. gingivalis*), and *Tannerella forsythia* (*T. forsythia*), in saliva and other internal surfaces of the oral cavity are associated with an increased risk of periodontal diseases [12,13,14,15]. Moreover, periodontal dysbiosis is also associated with the formation of pathogenic complexes consisting of anaerobic Gram-negative microbes. The orange complex is compounded with *Fusobacterium nucleatum* (*F. nucleatum*), *Prevotella intermedia*, *Prevotella micros*, and *Prevotella nigrescens*, shifting to the final form, the red complex, comprising *P. gingivalis*, *T. denticola*, and *T. forsythia*, and it appears during periodontal disease progression [16]. In turn, species such as *Actinomyces gerencseriae*, *Bifidobacterium*, *Streptococcus mutans* (*S. mutans*), *Veillonella*, and *Lactobacillus fermentum*, as well as *Streptococcus salivarius* (*S. salivarius*) and *Streptococcus parasanguinis* (*S. parasanguins*), have been associated with the development of early childhood caries [17,18].

**Figure 1:**
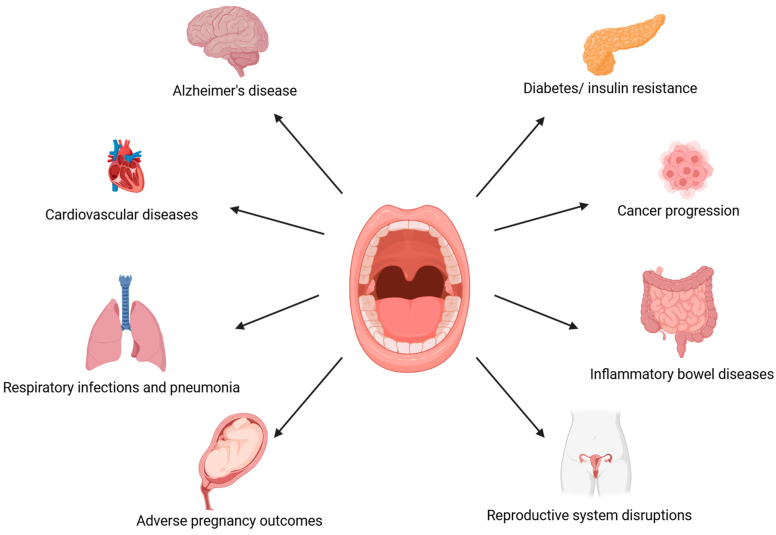
A potential link between oral dysbiosis and systemic health. (The figure was created using BioRender.com). Oral cavity ecological imbalance affects individual host systems. Periodontal diseases accompanied by oral dysbiosis are associated with the development of Alzheimer’s disease, cardiovascular disease, respiratory infections and pneumonia, adverse pregnancy outcomes, diabetes and insulin resistance, carcinogenesis, inflammatory bowel diseases, and disruptions in the reproductive system [21,22,23,24,25,26,27,28,29,30,31,32].

Recently, interest in the oral microbiome has increased due to the relationship between oral dysbiosis and the development of systemic diseases. Periodontal pathogens can induce the development of systemic diseases directly, via their transmission to distant organ systems through the bloodstream; or indirectly, by promoting low-grade inflammation due to the production of proinflammatory markers, such as interleukin (IL) 1, IL-6, or tumor necrosis factor (TNF-α) [19,20]. Injuries that damage blood vessels within dental plaque may enable the transmission of pathogenic bacteria via the bloodstream from the oral cavity to other host systems. Diseases associated with oral dysbiosis include Alzheimer’s disease, diabetes and insulin resistance, cardiovascular diseases, inflammatory bowel diseases, respiratory infections and pneumonia, and even some types of cancer (Figure 1) [21,22,23,24,25,26,27,28,29,30]. Oral microbiome dysbiosis may participate in the pathogenesis of these diseases and, at the same time, lead to dental caries and periodontal diseases. Recently, a connection has also been observed between the condition of the oral cavity and reproductive functions, as systemic inflammation induced by oral dysbiosis may affect fertility by lowering the chance of implantation [31,32].

The impact of oral health on female fertility is not yet fully understood, although some earlier reports suggest a certain relationship between the two. It is speculated that, similarly to the interaction of the oral microbiome with specific host systems, oral microbes may translocate from a dysbiotic oral cavity to the female reproductive organs, causing local inflammation. For instance, in a study by Nwhator et al. involving 70 pregnant women, the authors reported that poor oral hygiene and periodontitis were associated with an extended time to conception (>12 months) and suggested that periodontitis is positively correlated with a lower chance of conceiving [32]. However, although good oral hygiene was correlated with a shorter time to conception (<12 months) compared to poor oral hygiene, the difference was not statistically significant. Similar results were obtained in the SMILE study, which showed that among 146 women with a time to conception of >12 months, the incidence of periodontal disease was higher (34.9% vs. 25.7%) than in the group where the time to conception was shorter (<12 months) [33]. Women diagnosed with periodontal disease required an average of 7 months to conceive, whereas healthy patients conceived in an average of 5 months. In an observational and prospective study involving 256 non-pregnant women, researchers detected major periodontal pathogens in saliva and analyzed serum and saliva antibodies against these pathogens [34]. Participants underwent gynecological and clinical examinations to investigate a potential connection between periodontitis and conception. The follow-up period for becoming pregnant was 12 months. The study demonstrated that both salivary levels of *P. gingivalis* immunoglobulin A antibodies and the frequency of *P. gingivalis* in saliva samples were higher in the group of women who did not become pregnant, suggesting that *P. gingivalis* may be associated with conceive failure [34]. However, more research is still needed to confirm the connection between the oral and reproductive tracts and to understand this relationship.

The aim of this review was to discuss the composition of the female oral microbiota in relation to infertility-related disorders, such as endometriosis and polycystic ovary syndrome, and to describe the relationship between a dysbiotic oral microbiome, pregnancy, and its impact on the female reproductive tract. To provide a comprehensive overview of the current state of knowledge on this topic, the PubMed database was searched using the following keywords: ‘oral microbiome’ OR ‘oral microbiota’ OR ‘oral pathogens’ OR ‘oral microbes’ OR ‘oral microenvironment’ OR ‘oral health’ OR ‘mouth flora’ OR ‘periodontal health’ OR ‘periodontal disease’ OR ‘periodontitis’ OR ‘gingivitis’ AND ‘pregnancy’ OR ‘pregnancy outcomes’ OR ‘gestation’ OR ‘fertility’ OR ‘conception’ OR ‘implantation’ OR ‘reproductive health’ OR ‘reproductive tract’ OR ‘polycystic ovary syndrome’ OR ‘endometriosis’. When possible, the search was limited to the last 20 years and included only studies published in English. Both human and animal studies were included, and no restrictions were applied to the study design. Each publication was thoroughly evaluated. The authors first screened the titles and abstracts of potential articles, followed by a detailed review of the full texts to identify relevant studies. Any uncertainties or disagreements during the selection process were resolved by consensus.

## 2. Oral Health and Pregnancy Outcomes

Pregnancy is accompanied by very dynamic hormonal, immunological, and metabolic changes resulting from physiological alterations to establish fetal growth and development [35]. These alterations influence the mother’s microbiome of the gut, vagina, and oral cavity [36]. During pregnancy, there is an increase in the number of oral microorganisms toward dysbiosis, especially in the first trimester, which may be due to some *Prevotella* species, which are able to use estrogen and progesterone as substitutes for vitamin K for their growth [37,38,39,40]. Estrogen and progesterone metabolism are determined by the inflammatory component and the duration of inflammation, which influence the efficiency of enzymatic conversion within the gingiva [39,40]. Given that there are increased levels of sex steroid hormones in pregnancy, and they are able to be metabolized by localized in human gingiva-specific receptors and induce a local inflammation within, it is not surprising that oral diseases (such as gingivitis, dental caries, and periodontitis) are more prevalent in the gestational state, affecting about 40% of women worldwide [41]. Moreover, these gestational hormonal fluctuations affect the gingival tissues and cause inflammation, independent of the presence of proinflammatory cytokines such as IL-1 β or TNF-α in the gingival crevicular fluid (GCF) [42].

Although there is no established guideline for treating periodontal diseases among pregnant women, medical workers should pay more attention to oral hygiene education during pregnancy and raise awareness about dental care. According to the PERISCOPE longitudinal study among pregnant women in the first trimester, almost half of the participants (47.1%) had periodontitis, of which 66.7% had clinical signs, such as gingival bleeding [43]. Moreover, 41.3% of women declared that they had not been to the dentist at least one year before pregnancy, and 52.1% had poorer oral health, described as the presence of ≥10% dental plaque [43]. What is important is that the composition of the oral microbiome in pregnant women may change throughout pregnancy. For instance, Fujiwara et al. [38] demonstrated that in the first and second trimester, mouth flora show a predominance of *P. gingivalis* and *A. actinomycetemcomitans* compared to non-pregnant women, while *Candida* species were frequently abundant in the later stages of pregnancy. Moreover, it has been reported in a study by Massoni et al. [44] that there is a positive correlation between *P. gingivalis* and progesterone levels in the first trimester of pregnancy. During the first trimester of gestation, a higher abundance of *T. forsythia* in subgingival biofilm was observed, which increased susceptibility to the occurrence of gingivitis. Furthermore, Lin et al. [45] demonstrated that pregnant women are characterized by a higher abundance of *Treponema*, *Porphyromonas*, and *Neisseria*, as well as a reduced abundance of *Veillonella* and *Streptococcus*, compared to the non-pregnant group. The authors also demonstrated a significantly higher Shannon diversity index of the salivary microbiome in pregnant women compared to the non-pregnant group. In a study by Balan et al. [46], the authors examined bacterial communities of saliva and subgingival plaque during different stages of pregnancy and postpartum period. Using 16S rRNA sequence analysis, they aimed to determine the bacterial taxonomic differences among participants. In a group of pregnant women, the most abundant genera in saliva samples were *Prevotella*, *Veillonella*, *Streptococcus*, *Neisseria*, and *Terrahaemophilus*, while *Prevotella*, *Streptococcus*, *Fusobacterium*, *Veillonella*, and *Terrahaemophilus* predominated in subgingival plaque samples. Pregnancy was also associated with a higher abundance of pathogenic taxa at the species level, showing a predominance of *Prevotella* species, *P. gingivalis*, and *F. nucleatum*, which have been correlated to pregnancy gingivitis. Interestingly, the oral microbiome of pregnant women, which was characterized by an overgrowth of pathogenic bacteria, appeared to re-establish and form a healthy microbial community during the postpartum period. These findings suggest that hormonal alterations during pregnancy cause perturbations in the oral microbial environment, promoting a shift toward the overgrowth of pathogenic taxa.

Disruptions in the oral microbiome among pregnant women may not only influence maternal periodontal health but also interact with the placenta and affect the fetus. The placental microbiome is thought to be established through hematogenous microbial translocation from the maternal mouth flora, as these environments exhibit similarities to each other [47,48,49]. According to the theory of bacterial dissemination, it is suggested that periodontal pathogens may spread through the bloodstream to the amniotic fluid, leading to chorioamnionitis [50]. Given that the placental microbiome profile is similar to the supragingival plaque, it has been further suggested that oral dysbiosis during pregnancy may cause adverse pregnancy outcomes, such as premature birth, low birth weight, miscarriages, or preeclampsia [51,52,53,54,55]. Oral microorganisms found in the human placental include *A. actinomycetemcomitans*, *P. intermedia*, *T. forsythia*, *T. denticola*, *Campylobacter rectus*, *Fusobacterium nucleatum*, and *Porphyromonas gingivalis*, of which the most prevalent pathogens, *P. gingivalis* and *F. nucleatum*, are associated with intrauterine infections [48,52]. Periodontal pathogens such as *Actinobacillus actinomycestemcomitans*, *F. nucleatum*, *P. gingivalis*, *P. intermedia*, *T. denticola*, and *T. forsythia* were found in the placentas of pregnant women with preeclampsia [56], with increased levels of *F. nucleatum* and *P. intermedia* in the subgingival biofilm among postpartum women also associated with low birth weight and preterm delivery [57]. Cooper et al. [58] profiled subgingival and placental microbiomes of 54 women with and without preeclampsia and periodontal disease. They reported a significant predominance of oral-origin bacteria, including *Veillonella*, *Gemella*, *Granulicatella*, *Fusobacterium*, *Haemophilus*, *Streptococcus*, and *Neisseria*, in the placentas of pregnant women with preeclampsia compared to those without the condition (53.8% vs. 19.0%, respectively). Furthermore, the authors observed the highest prevalence of these pathogens in women with both preeclampsia and periodontal disease (58.8%). Moreover, results from recent studies have shown a connection between maternal oral health and preterm birth, suggesting that periodontitis may increase the risk of preterm delivery [59,60]. In particular, placenta colonization by oral pathogens such as *P. gingivalis* and *F. nucleatum* is suspected to contribute significantly to preterm delivery [61,62]. Periodontal infections may also influence birth weight and serve as a clinically significant factor for the risk of low birth weight. In a case–control study involving 88 postpartum women who delivered either low-birth-weight or normal-weight infants, poorer oral health, including a higher number of deep periodontal pockets, was reported among mothers of low-birth-weight babies [63]. Notably, deeper periodontal pockets increase the surface area for exchange between the bacterial biofilm and the bloodstream [64]. Additionally, both low birth weight and preterm birth have been associated with *P. gingivalis*, as its presence correlates with higher periodontal parameters, including plaque index, probing depth, and clinical attachment loss, and is inversely related to birth weight [65]. Interestingly, Kothwiale et al. [66] demonstrated an association between the severity of periodontal disease and lower hemoglobin levels. Both severe anemia and periodontitis, when combined, may influence fetal development, potentially leading to preterm delivery and low birth weight. In summary, pregnancy may favor the development of periodontal dysbiosis, which is accompanied by local inflammation. Consequently, oral pathogenic microbes produce proinflammatory mediators such as IL-1, IL-6, and TNF-alpha, along with bacterial endotoxins, like lipopolysaccharides (LPSs), which may disseminate systemically and affect fetus development. Selected studies investigating the relationship between periodontal health and adverse pregnancy outcomes are summarized in Table 1.

**Figure 2:**
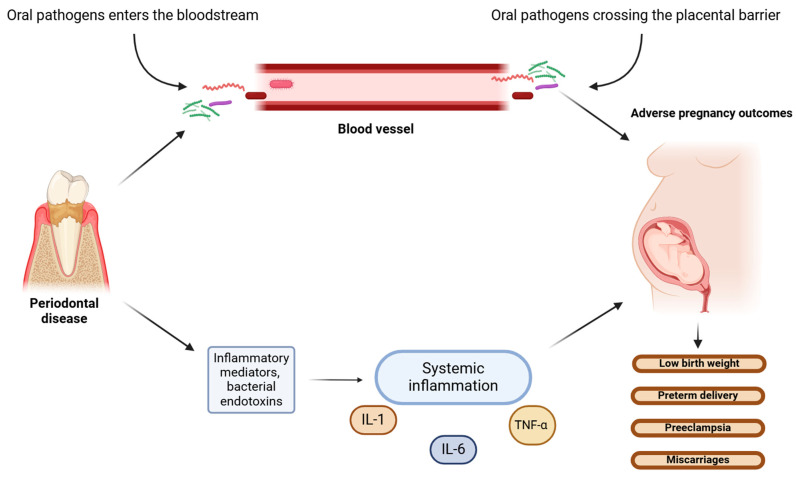
Potential direct and indirect mechanisms of oral pathogens’ actions on the fetus. (The figure was created using BioRender.com). Periodontal pathogens affect the fetus through two possible mechanisms: direct action, whereby microbes from disrupted oral microbiome translocate via hematogenous dissemination to the fetal circulation and cause bacteriemia; or indirect actions via oral bacterial endotoxins and inflammatory mediators entering the systemic circulation and causing systemic inflammation [67,70]. Systemic inflammatory response is induced by bacterial lipopolysaccharides (LPSs) and mediated by proinflammatory cytokines, including interleukins (IL-1 and IL-6) and tumor necrosis factor (TNF-α). Both mechanisms cause intrauterine and placental bacteriemia, leading to adverse pregnancy outcomes.

Animal studies are in line with these results, confirming the negative indirect effects of *P. gingivalis* on the placenta, such as fetal growth restriction caused by bacterial LPS [68]. *F. nucleatum*, when injected intravenously into pregnant mice, induced premature birth and stillbirths by spreading to the amniotic fluid and causing bacteriemia in the uterus, without leading to systemic dissemination [69]. These pathogenic species may induce adverse pregnancy outcomes via two possible mechanisms: directly, via hematogenous dissemination that translocates from the disrupted oral microbiome, crossing the placental barrier into the amniotic fluid and fetal circulation, consequently causing bacteremia; or indirectly, through endotoxins originating from the periodontium that enter the systemic circulation and affect the fetus (Figure 2) [67,70]. Furthermore, some oral commensal microorganisms associated with adverse pregnancy outcomes in humans may have a specific mechanism of translocation to the placenta, as there were different numbers of the microorganisms found in pregnant mice placentas than in injected saliva and subgingival plaque samples [49].

A meta-analysis of randomized controlled trials concluded that treatment of periodontal disease during pregnancy, such as scaling or root planning, may be beneficial for both the mother and the infant. Both of these dental medicine procedures reduce the rate of low birth weight (odds ratio (OR): 0.48; 95% confidence interval (CI): 0.23–1.00) and preterm delivery (OR: 0.55; 95%CI: 0.35–0.86), probably as a result of the reduced accumulation of pathogenic bacteria in the oral cavity and, consequently, decreased microbial transmission to the amniotic fluid [71]. Additionally, periodontal disease treatment might diminish inflammatory mediator levels in the systemic circulation, minimizing their negative effects on the reproductive tract. Professional periodontal care interventions during the second trimester are safe and may improve dental health by reducing gingival inflammation [72,73], thereby decreasing the negative effects of oral pathogens on the fetus. These findings underline the influence of periodontal disease accompanied by oral dysbiosis on maternal and neonatal health.

## 3. Oral Dysbiosis and Fertility Disorders

According to the World Health Organization (WHO) and the International Committee for Monitoring Assisted Reproductive Technology, infertility is a disease of the human reproductive system defined by the failure to achieve a clinical pregnancy within 12 months, despite regular sexual intercourse (2–4 times a week) without using contraceptive methods [74,75]. Infertility affects approximately 17.5% of the adult worldwide population, and at least 40 to 50% of the cases are caused by a male factor [75]. Female factors associated with infertility include mainly ovulation disorders caused by excessive stress; hormonal disorders; incorrect diet and lifestyle; and endocrine/gynecological disorders, e.g., polycystic ovary syndrome (PCOS) and endometriosis [76]. The most common gynecological disorders causing infertility in women, e.g., PCOS and endometriosis, are characterized by sex steroid hormone fluctuations and low-grade systemic inflammation. Given that female sex hormones may be used by some oral bacteria species, such as *P. intermedia* and *P. gingivalis*, as a source of nutrients, these conditions may increase the risk of periodontal disease [77].

### 3.1. Polycystic Ovary Syndrome

PCOS affects about 5–18% of women of reproductive age [78]. According to the Rotterdam criterion, this endocrine disorder is accompanied by the presence of at least two of the following: oligo- or anovulation, hyperandrogenism (clinical and/or biochemical), and polycystic ovaries [79]. Recent studies have shown a bidirectional relationship between PCOS and periodontal diseases, as these conditions are associated with low-grade systemic inflammation and may influence each other [80,81,82]. However, while this connection has been observed, a causal relationship between PCOS and periodontal diseases has not yet been established. Nevertheless, according to recent data, PCOS increases the risk of periodontal disease by 28%; in turn, periodontal disease increases the risk of PCOS by 46% [83]. PCOS may cause repercussions in the oral microbiome via elevated levels of proinflammatory markers such as TNF-α, C-reactive protein (CRP), IL-1, or IL-6, but also through the sex steroid hormone alterations, causing gingival inflammation [84,85,86]. Women with PCOS have been shown to exhibit elevated levels of estrogen and male androgenic hormones, along with decreased progesterone levels. These alterations in circulating hormones may affect female periodontal health [87]. Estrogen receptors are highly expressed in the gingival tissues of women with PCOS, with expression levels increasing 10-fold during inflammation [88,89]. The estrogen receptor subtype, ER-beta, is present in the gingival epithelium, salivary glands, and periodontal ligament [90,91,92]. Similarly, androgen receptors are found in human gingiva, and their metabolism in gingival tissues also increases during inflammation [93,94]. Collectively, elevated levels of estrogen and androgens may modulate gingival tissue and influence the oral microbiome in women with PCOS. In turn, periodontal disease promotes insulin resistance (IR), inflammation, and oxidative stress, risk factors for PCOS development [89]. Periodontal disease is accompanied by chronic inflammation and oxidative damage, which may increase the risk of PCOS by engaging anti-inflammatory and proinflammatory pathways. In studies by Özçaka et al. [85,86], higher concentrations of IL-6 were found in GCR, saliva, and serum of women with PCOS and gingivitis compared to those with PCOS but healthy periodontium [86]. Conversely, the second study reported decreased serum concentrations of IL-17E in PCOS women with gingivitis compared to the healthy group [85]. Moreover, proinflammatory mediators, such as IL-1, IL-6, and TNF-α, which are released during periodontal disease, play an essential role in the progression of IR [95]. Notably, *P. gingivalis*, which is highly abundant in the dysbiotic oral microbiome associated with periodontal disease, may exacerbate IR through a macrophage-dependent immune response in the oral cavity [96]. Subsequently, IR promotes the development of PCOS via its influence on pituitary luteinizing hormone (LH) secretion, resulting in increased androgen release by the ovaries [97].

A bidirectional relationship between PCOS and periodontal disease has been previously suggested in numerous studies [98,99,100]. In a study that enrolled 196 women (98 with PCOS and 98 healthy controls), evaluating periodontal parameters among both groups, a significantly higher rate of bleeding on probing (BOP) and clinical attachment loss (CAL) were reported in PCOS women in comparison to healthy subjects [101]. The authors observed increased frequency of periodontal disease in PCOS women, suggesting a bidirectional relationship between PCOS and periodontal disease, as both conditions are related to low-grade systemic inflammation and may exacerbate each other. Another study demonstrated a similar correlation between PCOS and periodontitis, as well as an associated periodontal prooxidative state, indicating that PCOS patients have a higher susceptibility to periodontitis [102]. Moreover, the prevalence and likelihood of periodontitis are significantly higher in women with newly diagnosed PCOS [103]. These women also exhibit higher levels of periodontal inflammation and tissue breakdown compared to medically treated PCOS women and healthy controls. Additionally, the same study has also shown an association between periodontal breakdown and systemic inflammation, highlighting the role of oral health in modulating systemic inflammatory pathways. Akcali et al. [104] reported a correlation between PCOS and microbial oral dysbiosis, demonstrating that this hormonal disorder may interfere with the oral microbiome composition. Increased salivary levels of putative periodontal pathogens were observed in women with PCOS and gingivitis, such as *P. gingivalis*, *T. forsythia*, and *F. nucleatum*, compared to the periodontally healthy controls. A particularly strong effect was observed in the case of *P. gingivalis*, as PCOS increased the occurrence of this pathogen in saliva, as well as the serum antibody response, especially in the presence of gingivitis. Moreover, PCOS also selectively increased the serum antibodies to *P. intermedia* and *S.oralis* [104]. The presence of *F. nucleatum*, *T. forsythia*, *P. gingivalis*, and *P.intermedia* in the subgingival plaque is positively associated with periodontal inflammation progression [105]. Similar results among participants with PCOS and periodontitis and patients with PCOS and gingivitis were also observed in a case–control study of 40 women which demonstrated that levels of *F. nucleatum* and *P. gingivalis* in the subgingival plaque are higher in women with PCOS compared to healthy controls [80]. Interestingly, Li et al. [106] observed that PCOS patients have disrupted diurnal rhythm of the salivary microbiome and that their microbial composition may change during the day. Participants with PCOS showed differences in alpha and beta diversity at two time points compared to healthy controls with a higher salivary abundance of *Fusobacterium* at each time point, as well as a lower abundance of *Actinobacteria* [106]. Moreover, there was a reduced abundance of *Actinobacteria* in saliva observed in a group of periodontally healthy PCOS women [107]. The reduced abundance of *Actinobacteria* has been associated with periodontitis [108,109], confirming that PCOS patients have a more pathogen-favorable microenvironment, which may involve a higher susceptibility to the development of periodontal diseases affecting future gestation.

Although a connection between oral microbes and PCOS has been documented in earlier studies, there is still limited research on the influence of dental treatment on systemic health among women with PCOS. However, a randomized controlled trial by Deepti et al. [110] investigated the impact of non-surgical periodontal treatment combined with medical therapy using Myo-inositol on serological inflammatory markers, including high-sensitivity C-reactive protein (hsCRP) and insulin resistance (assessed by Homeostatic Model Assessment; HOMA-IR), among 60 women with PCOS and periodontitis who were randomly divided in two groups. Participants in both groups received Myo-inositol at a dose of 2 g per day for 6 months. In addition, women in the test group underwent 2–3 sessions of periodontal treatment, including root planning, scaling, and oral hygiene instructions. The control group received education on proper oral hygiene and information about periodontal disease but did not receive any dental therapy. Serum levels of hsCRP decreased in both groups; however, the reduction was statistically greater in the test group. The authors also reported a significant improvement in periodontal parameters in the test group, including reduced gingival inflammation, fewer sites with bleeding on probing, lower plaque levels, and decreased pocket depth. These results suggest that non-surgical periodontal treatment, such as root planning and mouth scaling, combined with medical therapy, may be beneficial in reducing low-grade systemic inflammation among patients with PCOS [110]. Moreover, it has been documented that the treatment of periodontal disease can be as effective in reducing CRP levels as pharmacological treatment or lifestyle modifications [111]. Considering that women with PCOS exhibit significantly higher levels of pro-inflammatory cytokines in saliva [112], it is plausible to suggest that dental treatments aimed at improving oral health may also help alleviate the symptoms of this hormonal disorder.

### 3.2. Endometriosis

Endometriosis is a chronic gynecological disease with an inflammatory component and the presence of endometrium-like epithelium outside the endometrium and myometrium [113]. Due to the often asymptomatic nature of the disease and difficulties in its diagnosis, it is impossible to determine the exact frequency of its occurrence, but, according to estimates, it may affect up to 50% of infertile women [114]. This condition is asymptomatic in about 20–25% of females, known as ‘silent endometriosis’, although typical symptoms include chronic pelvic pain, dysmenorrhea, infertility, deep dyspareunia, severe menstrual pain, etc. [115]. There is a hormonal imbalance in endometriosis, e.g., increased estrogen levels, in combination with insufficient progesterone [116].

A growing body of research demonstrates the association between the development of endometriosis and the gut microbiome. Studies have shown that the gut microbiome in endometriosis is characterized by an overgrowth of *Proteobacteria*, *Verrucomicrobia*, *Fusobacteria*, and *Actinobacteria*, with a dominance of *Shigella* and *Escherichia*, as well as significantly decreased *Lactobacillaceae* [117,118,119,120,121,122]. The collection of genes in the gut microbiome, which encode estrogen-metabolizing enzymes, is described as the estrobolome, and it regulates estrogen levels [123]. Given that, perturbations in the gut microenvironment may disrupt circulating estrogen levels and induce estrogen-dependent diseases [123,124]. A difference in microbial composition in subjects with endometriosis was also shown in the peritoneal fluid and female reproductive tract [118,125,126,127,128]. These alterations in the microbiome, promoting dysbiosis, play an important role in the pathogenesis of endometriosis by modifying circulating estrogen levels or inducing systemic inflammation by pathogenic bacteria [129].

**Figure 3:**
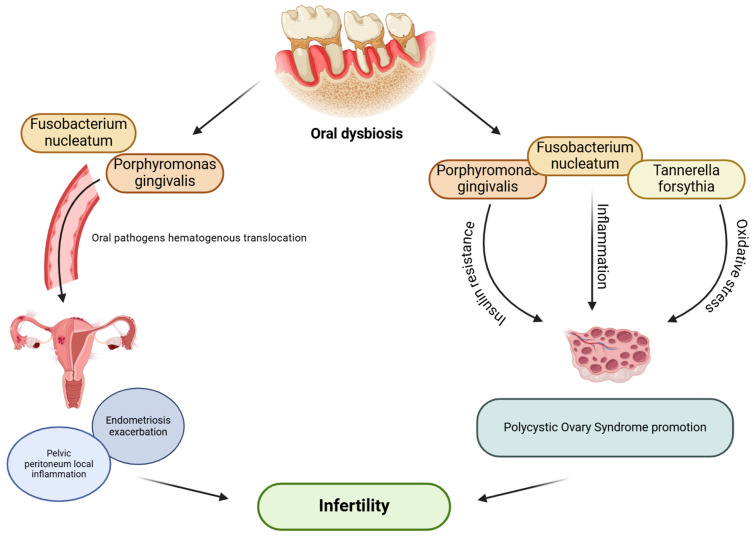
Possible effect of oral dysbiosis on female infertility. (The figure was created using BioRender.com). Oral dysbiosis and periodontal pathogens associated with it, such as *F. nucleatum*, *P. gingivalis*, and *T. forsythia*, may translocate from disrupted oral microenvironment and affect female reproductive functions; directly, via inducing local inflammation in the pelvic peritoneum among women with endometriosis, causing exacerbation of the disease; or indirectly, via promoting inflammation, insulin resistance, or oxidative stress, which may lead to PCOS development [84,85,86,89,104,137,139].

There is a lack of research regarding the connection between endometriosis and oral microbiome, although a few previous studies showed an association between periodontitis and endometriosis [130,131]. Recently, patients with endometriosis and repeated implantation failure have been shown to have elevated levels of *Dialister* and *Streptococcus* in the uterine endometrium microbiome, which, along with a higher Shannon diversity index in the uterine endometrium microbiome, was suggested to be associated with the pathology of constant implantation failure in endometriosis patients [132]. Importantly, the Gram-negative *Dialister* species occurs in the oral cavity and has been previously associated with periodontal diseases [133]. *D. pneumosintes* is more frequently prevalent in the subgingival biofilm among subjects with periodontitis [133,134,135,136] and is also detected in the placenta of women with preeclampsia [136]. Jin et al. [137] proved for the first time a bidirectional causal effect between periodontitis and endometriosis, demonstrating that periodontitis has a cause–effect on endometriosis of the pelvic peritoneum. The authors also proposed a few mechanisms to explain the cause–effect of periodontitis on endometriosis of the pelvic peritoneum. Periodontal pathogens associated with dysbiosis, such as *P. gingivalis*, may translocate from the oral cavity to the pelvic peritoneum and induce local inflammation via activating peritoneal macrophages and interleukin-1 synthesis. A study by Young et al. [138] emphasized the crucial role of the pelvic peritoneum in the development and maintenance of endometriosis. Furthermore, recent data from an in vivo mice study by Muraoka et al. [139] demonstrated that *F. nucleatum* infection causes macrophage infiltration in the endometrium and promotes endometriotic lesion development. *F. nucleatum* was detected in the uterus of 64% of patients with endometriosis and may be related to endometriosis progression. As mentioned before, intrauterine infections may be induced by *F. nucleatum* due to its transmission from the oral cavity through the hematogenous system to the placenta [140]. It is hypothesized that the same transmission of pathogenic microbes from the oral cavity to the uterus occurs in patients with endometriosis who are more susceptible to oral dysbiosis and are at risk of periodontal disease. Consequently, the infiltration of *F. nucleatum* into endometrial tissues contributes to disease exacerbation by promoting local inflammation. These observations suggest that oral health may significantly influence the progression and course of endometriosis, as well as successful implantation (Figure 3).

## 4. Probiotics Administration in Fertility Disorders

In recent years, probiotics supplementation has emerged as a promising method to improve fertility [141]. *Lactobacillus* strains are of particular interest, as they might support women’s reproductive health by displacing pathogenic species and producing lactic acid within the endometrial or vaginal flora [142,143,144]. Vaginal administration of probiotics has been proposed to be more effective than oral delivery methods [145]. However, there is a lack of research examining their impact on fertility in women with periodontitis, and data regarding the influence of probiotics on periodontal disease are limited and insufficient to clearly state their benefits [146,147]. Nevertheless, probiotic administration has shown beneficial effects in PCOS patients, improving metabolic parameters crucial for fertility, including HOMA-IR, CRP and total testosterone levels, all of which are correlated with periodontitis [148,149,150]. Unfortunately, there are still limited data on the use of probiotics in women with endometriosis, although two studies have shown positive effects [151,152]. Further investigations are needed to assess the impact of the use of antibiotics or probiotics on the risk of developing both PCOS and endometriosis.

## 5. Conclusions and Future Perspectives

The human oral microbiome impacts systemic health, including the female reproductive tracts and reproduction. Periodontal pathogens may translocate from the oral cavity to the uterus and can also migrate to the placenta via the hematogenous system. This microbial transmission perturbs the genital microbiome, leading to local inflammation and functional impairment. Moreover, this link between oral health and reproductive functions seems to be bidirectional, as some conditions of the reproductive tract, accompanied by hormonal imbalance and/or ongoing inflammation, create a favorable environment for the development of oral dysbiosis. Disease exacerbation among women of reproductive age may consequently lead to implantation failures or even gestational complications. According to a growing body of research on the oral–reproductive connection, the oral microbial community should be considered as one of the factors influencing female fertility. The overgrowth of pathogenic bacteria in oral cavity affects the female fertility either directly, via bacterial translocation from the oral cavity to the reproductive system via the bloodstream; or indirectly, through the production of proinflammatory mediators. This relationship is particularly notable for *F. nucleatum* and *P. gingivalis*, which are associated with adverse pregnancy outcomes, as well as common causes of infertility, such as PCOS and endometriosis.

There are still insufficient data to conclusively determine whether supplementing with probiotics or improving oral health can significantly impact fertility and future conception. Some studies suggest that probiotics administration may help improve health parameters associated with periodontal disease, such as HOMA-IR, CRP, or testosterone levels in women with PCOS. Future research should focus on assessing whether increasing dental health through probiotics, antibiotics, or better oral hygiene might reduce the risk of PCOS, endometriosis, and pregnancy issues. Improving periodontal health through periodontitis treatment may reduce systemic inflammatory responses and increase the chances of successful implantation. Therefore, maintaining good oral hygiene should be a priority for every woman planning a pregnancy, particularly for those at increased risk of periodontal disease.

These insights into the relationship between the female reproductive tract and oral health emphasize the significance of periodontal health among women trying to conceive. Therefore, it is recommended that women preparing for pregnancy be surrounded by professional gynecological, as well as dental healthcare. Enhancing oral hygiene may improve reproductive health by reducing the inflammatory systemic response and alleviating disease symptoms. Future studies should explore the association between periodontal health and female fertility to inform the development of guidelines for women of reproductive age affected by infertility and during pregnancy. Dental prevention strategy may increase the chances of conception and reduce the risk of complications during gestation.

## Figures and Tables

**Table 1:** Summary of selected studies on the association between oral health and adverse pregnancy outcomes.

Study	*n*	Study Design	Investigated Pregnancy Outcome	Results
Moore et al. [52] (2004)	*n* = 3738	Prospective study	Preterm birth Low birth weight Miscarriage	Poorer periodontal health was associated with an increased risk of late miscarriage; however, no significant relationships between the severity of periodontal disease and preterm birth or low birth weight were shown.
Farrell et al. [53] (2006)	*n* = 1793	Prospective study	Preterm birth Low birth weight Miscarriage	No association between periodontitis and preterm birth or low birth weight among never-smokers was found; however, late miscarriage was associated with some measures of periodontal disease, like higher mean probing depth at mesial sites.
Barak et al. [56] (2007)	Cases: *n* = 16 Controls: *n* = 14	Case–control study	Preeclampsia	A significant presence of periopathogenic microorganisms (*A. actinomycetemcomitans*, *F. nucleatum* ssp., *P. gingivalis*, *P. intermedia*, *T. forsythensis*, and *T. denticola*) or their products was detected in human placentas of women with preeclampsia.
Katz et al. [61] (2009)	Cases: *n* = 9 Controls: *n* = 5	Case–control study	Preterm birth	The presence of *Porphyromonas gingivalis* antigens in placental tissues was associated with preterm delivery.
Chen et al. [65] (2012)	Cases: *n* = 72 Controls: *n* = 38	Retrospective study	Low birth weight	A significantly lower birth weight, as well as higher plaque index, probing depth, and clinical attachment loss, were observed in the *P. gingivalis*-positive group.
Haerian-Ardakani et al. [63] (2013)	Cases: *n* = 44 Controls: *n* = 44	Case–control study	Low birth weight	An association was found between a greater number of deep periodontal pockets, poorer maternal oral health, and low birth weight.
Kothwiale et al. [66] (2014)	*n* = 770	Observational study	Preterm birth Low birth weight	Periodontitis was significantly associated with low birth weight, and the increase in the severity of periodontal disease was associated with a higher rate of preterm birth.
Perunovic et al. [67] (2016)	Cases: *n* = 60 Controls: *n* = 60	Cross-sectional study	Preterm birth	Poorer oral health, higher rate of periodontitis, and significantly higher levels of proinflammatory cytokines (IL-6 * and PGE2 **) in GCF *** were observed among women with preterm birth.
Ye et al. [62] (2020)	Cases: *n* = 28 Controls: *n* = 36	Longitudinal study	Preterm birth	The presence of *F. nucleatum* in placental tissues was significantly associated with a threatened preterm birth.
De Oliveira [60] (2021)	*n* = 2474	Cohort study	Preterm birth	Periodontal disease increased the risk of early preterm delivery, making it almost two times higher.
Shaggag et al. [59] (2022)	*n* = 165	Case–control study	Preterm birth	A strong association was found between periodontitis and low hemoglobin levels with preterm birth among women with spontaneous preterm delivery.
Lima et al. [57] (2023)	Cases: *n* = 40 Controls: *n* = 80	Case–control study	Preterm birth Low birth weight	A possible relationship was observed between periodontitis and preterm birth, as well as low birth weight, based on clinical parameters and the ratio of *P. intermedia* and *F. nucleatum* at periodontal sites.
Cooper et al. [58] (2024)	*n* = 54	Prospective, cross-sectional cohort study	Preeclampsia	A significant presence of a combined group of oral-origin bacteria was detected in the placentas of pregnant women with preeclampsia.

*** GCF, gingival crevicular fluid; * IL-6, interleukin-6; ** PGE2, prostaglandin E2.

## Data Availability

No new data were created or analyzed in this study.
